# A Link Prediction Algorithm Based on Weighted Local and Global Closeness

**DOI:** 10.3390/e25111517

**Published:** 2023-11-06

**Authors:** Jian Wang, Jun Ning, Lingcong Nie, Qian Liu, Na Zhao

**Affiliations:** 1 Faculty of Information Engineering and Automation, Kunming University of Science and Technology, Kunming 650500, China; jianwang@kust.edu.cn (J.W.); 20222204358@stu.kust.edu.cn (J.N.); 20212204158@stu.kust.edu.cn (L.N.); 2 Yunnan Key Laboratory of Artificial Intelligence, Kunming University of Science and Technology, Kunming 650500, China; 3 School of Software, Yunnan University, Kunming 650091, China; liu_antoni0409@163.com; 4 School of Management, Harbin Institute of Technology, Harbin 150001, China

**Keywords:** complex network, link prediction, cluster coefficient, node proximity

## Abstract

Link prediction aims to identify unknown or missing connections in a network. The methods based on network structure similarity, known for their simplicity and effectiveness, have garnered widespread attention. A core metric in these methods is “proximity”, which measures the similarity or linking probability between two nodes. These methods generally operate under the assumption that node pairs with higher proximity are more likely to form new connections. However, the accuracy of existing node proximity-based link prediction algorithms requires improvement. To address this, this paper introduces a Link Prediction Algorithm Based on Weighted Local and Global Closeness (LGC). This algorithm integrates the clustering coefficient to enhance prediction accuracy. A significant advantage of LGC is its dual consideration of a network’s local and global features, allowing for a more precise assessment of node similarity. In experiments conducted on ten real-world datasets, the proposed LGC algorithm outperformed eight traditional link prediction methods, showing notable improvements in key evaluation metrics, namely precision and AUC.

## 1. Introduction

Complex network research has progressively become a focal point in scientific domains, providing a robust framework for exploring the structure and functionality of various real-world systems. Specifically, link prediction, as a key branch, exhibits irreplaceable value in social networks [1,2,3], biological networks [4], and information dissemination domains [5,6,7]. The goal of this research is to predict potential or missing links based on the known network topology and attributes, thereby offering insights into network evolution, forecasting future relations between nodes, and optimizing resource and information allocation and dissemination.

Despite significant progress in network science across various fields, link prediction remains a vibrant research area, partly due to real-world networks being dynamic, large-scale, and structurally intricate. The key challenges include accurately and efficiently calculating node similarity in a vast potential edge space, ensuring scalability of prediction methods in large networks [8], and mining meaningful patterns from known topologies to predict unknown links.

Traditionally, link prediction methods predominantly rely on node similarity measures, which vary in granularity, including local information-based, path-based, and random-walk-based approaches [9,10,11]. For instance, the Common Neighbor (CN) [12,13] index is one of the earliest and simplest similarity indices based on local information. Adamic et al. introduced the Adamic–Adar (AA) [14] index by considering the individual differences in CN. Zhou et al. proposed the Resource Allocation (RA) [15,16] index by referring to the resource allocation process in complex networks. The Preferential Attachment (PA) [17] index suggests that the probability of link formation is proportional to the product of the degrees of the two nodes. The CCLP algorithm [18] takes into account the clustering coefficient of common neighbor nodes and proposes a link prediction algorithm based on the clustering coefficient. The NDCC link prediction algorithm [19,20] defines the similarity between nodes as the combined effect of node degree and clustering coefficient. In path-based methods, the Katz centrality metric [19,20] considers all paths and assigns greater weights to shorter paths while giving smaller weights to longer paths when computing the contribution of path weights as a similarity metric. Additionally, the Local Path (LP) [21] metric is a comprehensive metric that combines path information based on common neighbors, and the LP metric extracts path information of third-order neighbors between the target nodes as a similarity metric [22,23,24]. In the realm of methods based on random walks, García-Pérez and colleagues [25] employed n-chain iterative algorithms and tensor graph-based random walk algorithms to enhance node similarity measurements and predictive performance across various networks. Despite these methods showing excellent accuracy and speed, most are supervised and require prior training, entailing high costs [26,27]. These efforts have enriched the field of link prediction, yet they underscore the ongoing reliance on node similarity, highlighting the need for a multifaceted approach.

However, the existing classic algorithms such as CN, AA, RA, PA, and LP only consider the degree or count of common neighbor nodes. These algorithms have some issues, such as low accuracy in predicting results. The CCLP index and the four evaluation indices CN, AA, RA, and PA have the opposite focus. They only consider the influence of the clustering coefficient in similarity indices, overlooking the important attribute information contained in node degree. The NDCC algorithm combines the clustering coefficient and node degree. However, these algorithms still have the problem of not considering node closeness centrality and not fully exploring the global information of the network. In light of the above aspects, this paper proposes a Link Prediction Algorithm based on Weighted Local and Global Closeness, abbreviated as LGC. The contributions of this work are as follows: (1) introduction of inter-node closeness based on node closeness; (2) proposal of local attribute closeness and global attribute closeness; (3) development of a link prediction algorithm weighted by local and global closeness; (4) optimization of this algorithm to yield improved performance; (5) validation of its effectiveness through experiments on ten real-world datasets.

This paper is organized as follows: we introduce the problems in link prediction and baseline algorithms and present the LGC algorithm in Section 2. In Section 3, we describe the experiments and analysis of the results of the proposed methods. In Section 4, we provide the overall conclusion of the work.

## 2. Algorithm Description

### 2.1. Problem Description

Consider an undirected, unweighted simple graph $G\left(V,E\right)$, where  E represents the set of nodes in the network, and V  represents the set of edges. We can define a universal set U that contains all possible pairs of N nodes, and all possible combinations of nodes that could potentially form an edge. The core problem of link prediction is that, based on the given graph structure and a certain algorithm, we must calculate a link probability for those pairs of nodes that are not directly connected in the graph G. Let $S_{xy}$ be defined as the similarity measure between node x and node y. The magnitude of this measure is positively correlated with the probability that a link will be formed between these two nodes. For all $S_{xy}$, if we sort them in descending order, then the pairs of nodes with higher ranks are more likely to form a link in the graph.

### 2.2. Classical Algorithm Similarity Metric

The symbols used in this paper and the definitions of similarity concerning classical algorithms are presented in Table 1 and Table 2.

### 2.3. LGC Algorithm and LGC* Algorithm

#### 2.3.1. Node Closeness

**Figure 1:**
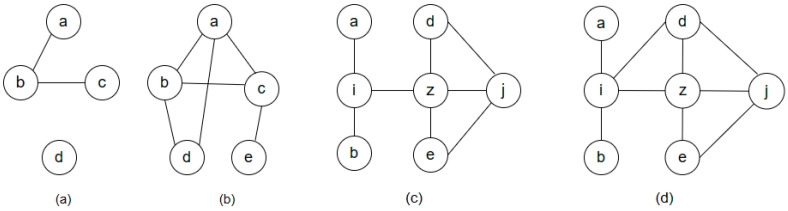
Two sets of networks explaining closeness centrality, where (**a**,**b**) form the first set of networks, and (**c**,**d**) form the second set of networks.

In scenarios such as information dissemination, social influence, disease propagation, and logistics management, a node’s ability to rapidly reach other nodes within the network, known as its closeness, is crucial to its role. Therefore, the introduction of node closeness allows for a more comprehensive and in-depth understanding of the structure and dynamics of complex networks. Consider the simple network examples in Figure 1. Nodes a, b, and c in network Figure 1a form a chain-like structure. Their clustering coefficient is 0, as there are no additional connections between their neighbors. The closeness centrality of nodes a and c is low because they are connected via node b. Due to the low clustering coefficients of nodes a, b, and c, this metric cannot be used to predict new links forming between them. 

In network Figure 1b, nodes a, b, and c form a triangular structure, resulting in a higher clustering coefficient. There is a direct connection between node a and node d; hence, the closeness centrality of a and d is relatively high. Because nodes a, b, and c have a high clustering coefficient, it is possible to predict that new links may form between a and b, or between b and c in the future. In addition, due to the high closeness centrality between nodes a and d, it is also possible to predict that a new link may form between them. 

The rationale for introducing node closeness centrality in this paper lies in its ability to measure the importance of a node in a network from a different perspective. While the degree of a node (the number of edges directly connected to it) can provide some information, we desire a deeper understanding of a node’s role within the network, beyond merely the quantity of its direct neighbors.

Compared to network (c) in Figure 1, nodes i and d in the network Figure 1d are now connected, resulting in a reduction in the average shortest path length between nodes i and d. With more paths from node i to node j in network Figure 1d, the likelihood of nodes i and j being connected increases. Closeness centrality takes into account the average distance of a node to other nodes along the shortest path. The higher the closeness centrality of a node, the more likely previously unconnected nodes are to be linked to it. Closeness centrality for a node is defined as follows:

**Definition** **1.** *Closeness Centrality (* C *): For any given node* i *in the network, its closeness centrality is defined as follows:*

(1) $$C\left(i\right)=\frac{1}{d_{i}}=\left(n-1\right)/\underset{j\neq i}{\sum }d_{ij}$$

*where* $C\left(i\right)$ *represents the closeness centrality of a network node* i *, and* $d_{ij}$ *is defined as the average distance to all other nodes in the network.*

In link prediction, we are concerned with the establishment of a connection status between two entities. By incorporating Formula 1, we can regard pairs of nodes as a small unit, thereby defining a proximity centrality for node pairs. The objective is to transition from the retrieval of information about an individual node to the retrieval of information about a pair of nodes.

**Definition** **2.** *In a network, for any two arbitrary nodes* i *and* j *, the nodal closeness between these nodes is defined as follows:*

(2) $$C\left(i,j\right)=C\left(i\right)+C\left(j\right)$$

#### 2.3.2. Local Closeness and Global Closeness

To enhance the accuracy of predictions, researchers typically consider various properties in the network. These properties are generally categorized into local and global attributes. Local attributes refer to properties directly related to individual node pairs, such as the number of mutual friends between two people in a social network, which can be regarded as a local attribute. Additionally, attributes like the degree of a node and the shortest path length between two nodes are also considered local. On the other hand, global attributes reflect the overall structure or properties of the entire network, such as the network’s diameter and average clustering coefficient. Local attributes are especially useful for predicting nodes that are adjacent or strongly connected, while global attributes are more crucial for link prediction tasks influenced by the overall network structure. Hence, combining both local and global attributes often yields better results.

Traditionally, closeness is understood as a local attribute because it is defined based on an individual node and its position within the graph. However, it does indeed reflect the relationship of the node with the rest of the network. From this perspective, we can introduce a new classification for closeness: local closeness and global closeness.

**Definition** **3.** *Local Attribute Closeness: For any two arbitrary nodes* i *and* j *in a network, the closeness of their local attributes is defined as follows:*

(3) $$LC\left(i,j\right)=\underset{z\in \Gamma \left(i\right)\cap \Gamma \left(j\right)}{\sum }\frac{C\left(i,j\right)+CC\left(z\right)}{k_{z}}$$

*where* z *represent the first-order common neighbors of nodes* i *and* j*,* $CC\left(z\right)$ *represent the clustering coefficient of node* z*, and* $k_{z\;}$*represent the degree of the node* z.

To obtain more accurate predictions, we also need global information from the network. The network average clustering coefficient measures the tightness of nodes within the network. A higher average clustering coefficient means that nodes in the network are inclined to group together. This typically indicates more frequent information transmission, interaction, and collaboration within the network. 

The network average shortest path length, on the other hand, refers to the average of the shortest path lengths between all pairs of nodes in the network. A shorter average shortest path length enhances the speed of information and resource propagation within the network. Based on the combination of the above two aspects, we propose Global Attribute Closeness.

**Definition** **4.** *Global Attribute Closeness: For any two nodes* i *and* j *in the network, the global attribute closeness is defined as follows:*

(4) $$GC\left(i,j\right)=\frac{C\left(i,j\right)d}{CC}$$

*where* CC *represents the average clustering coefficient of the network and* d *represent the average shortest path length of the network.*

#### 2.3.3. Link Prediction Algorithm Based on Weighted Local and Global Closeness (LGC)

**Definition** **5.** *For an undirected and unweighted simple network* $G\left(V,E\right)$ *, the similarity of the predicted node pair* $\left(x,y\right)$ *is defined based on the Local and Global Consistency Weighted Prediction Index (LGC).*

(5) $$S_{xy}^{LGC}=\lambda \underset{z\in \Gamma \left(x\right)\cap \Gamma \left(y\right)}{\sum }\frac{C\left(x,y\right)+CC\left(z\right)}{k_{z}}+\left(1-\lambda \right)\frac{C\left(x,y\right)d}{CC}$$

*where* λ *is an adjustable parameter used to adjust the balance between local information and global information.*

In multiple experiments, an optimized algorithm (LGC*) was proposed based on the aforementioned algorithm (LGC).

**Definition** **6.** *For an undirected and unweighted simple network* $G\left(V,E\right)$ *, the optimized similarity of the predicted node pair* $\left(x,y\right)$ *is defined as:*

(6) $$S_{xy}^{LGC^{*}}=\lambda \underset{z\in \Gamma \left(x\right)\cap \Gamma \left(y\right)}{\sum }\frac{{\left(C\left(x,y\right)+CC\left(z\right)\right)}^{2}}{k_{z}}+\left(1-\lambda \right)\frac{C\left(x,y\right)d}{CC}$$

**Figure 2:**
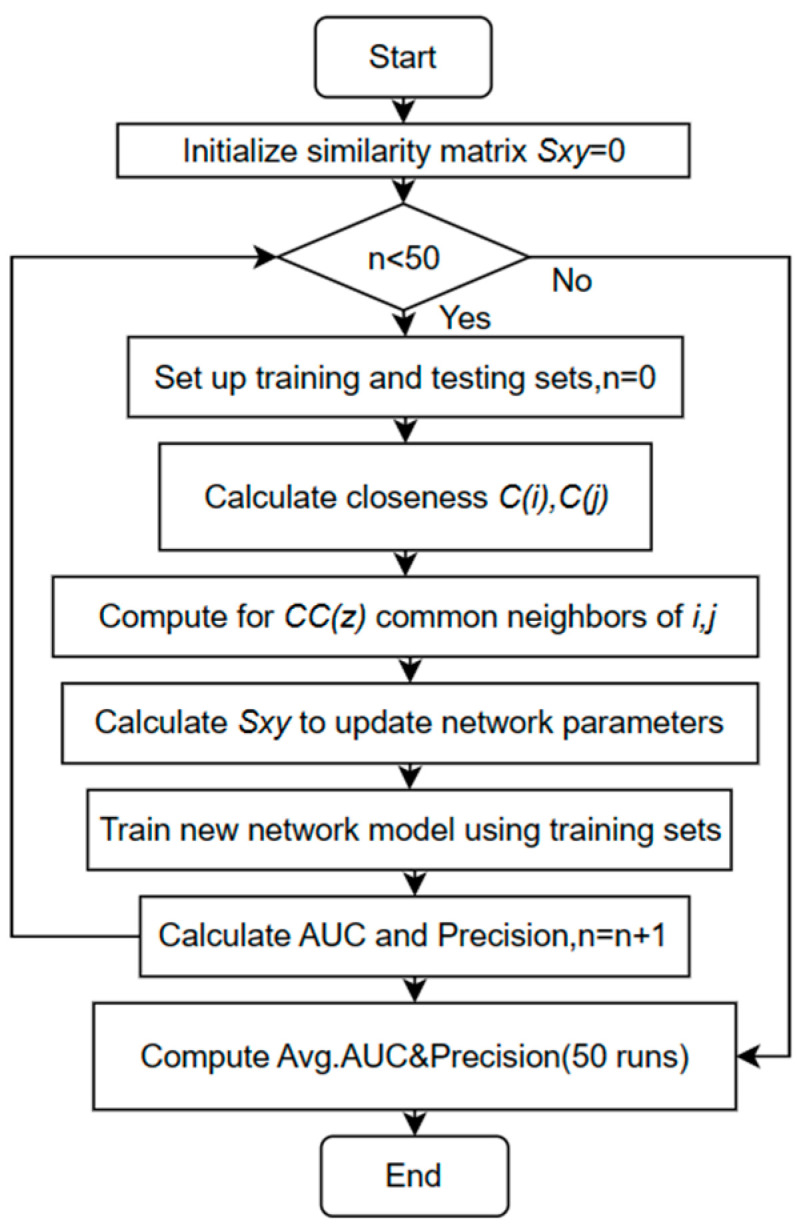
LGC algorithm flowchart.

Figure 2 shows the flowchart of the LGC algorithm (the algorithm flow of LGC* is consistent with LGC. The difference lies in the calculation of similarity scores according to the above formula).

## 3. Results and Analysis

In the network, the set of all edges E is divided into two distinct parts: the training set $E_{T}$ and the test set $E_{P}$. A suitable ratio is specified for this division, commonly set at 9:1 for the training set to the test set. There are no overlapping edges between the two sets, namely, $E_{T}\cup E_{P}=E$, $E_{T}\cap E_{P}=\emptyset$.

### 3.1. Datasets

To validate the effectiveness of the algorithm, experiments were conducted on the following ten real-world network datasets:

USAir, a network constructed from airline routes between airports in the United States;

PolBooks, a network formed from books related to American politics sold by an online bookstore;

CE, a network formed from the connections between neurons in nematodes;

LESM, a network constructed from characters that appear together in the novel Les Misérables;

JAMA, a network representing the social relationships between Japanese macaques;

Jazz, a network derived from collaborative relationships between jazz musicians;

Route Network, a network formed from the traffic paths between key locations (such as cities or transport hubs) in a certain transportation network;

Football, a network constructed from the game relationships between American college football teams in a season;

Karate Club Network, a network formed from the social relationships among members of a karate club;

STMA, a network constructed from the interactions between species.

In the statistical Table 3, *N* represents the number of nodes in the network, *M* represents the number of edges, *<c>* represents the average clustering coefficient, *<k>* represents the average degree, and *D* represents the density of the network. In subsequent experiments, each data testing metric is the mean value of 50 experimental results.

### 3.2. Evaluation Metrics

The metric of AUC (Area Under the Curve) represents the probability that a randomly chosen positive sample (a pair of nodes that exist in the test set $E_{P}$) has a higher similarity score than a randomly chosen negative sample (a pair of nodes that do not exist in the test set $E_{P}$). In the context of link prediction, positive samples typically refer to the pairs of nodes that exist in a graph (the edges), while negative samples refer to the pairs of nodes that do not have an edge between them in a graph. The goal of link prediction algorithms is to compute a similarity score for pairs of nodes in the test set $E_{P}$ based on the existing connection information in the graph (usually provided by the training set $E_{T}$). For evaluation, each time we randomly selected a pair of nodes that actually exist in the test set $E_{P}$ (positive sample) and a pair of nodes that do not exist (negative sample). We compared the similarity scores of these two sets of node pairs. Based on the comparison results, we recorded the counts of the following three situations:

$N_{1}$: the number of times the similarity score of positive samples in the test set is greater than that of negative samples;

$N_{2}$: the number of times the similarity score of positive samples in the test set is equal to that of negative samples;

$N_{3}$: the total number of comparisons made.

(7) $$\begin{array}{c} AUC=\frac{N_{1}+0.5N_{2}}{N_{3}} \end{array}$$

The precision metric calculates the proportion of actual accurately predicted *n* pairs of nodes (pairs of nodes that truly have a connection) among the top m pairs of nodes that have the highest similarity scores in the test set, as computed by the link prediction algorithm. The precision metric is defined as follows:

(8) $$\begin{array}{c} Precision=\frac{n}{m} \end{array}$$

### 3.3. Analysis of Results 

**Figure 3:**
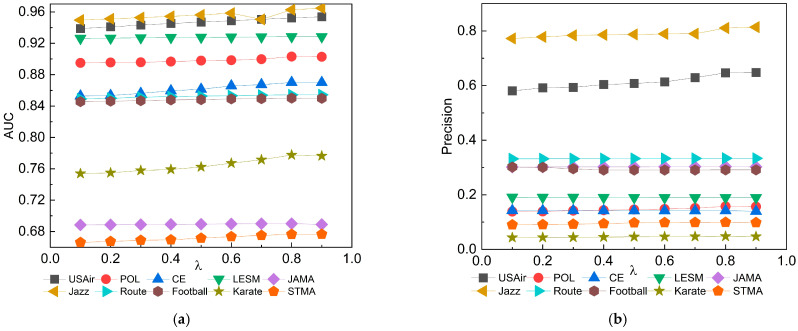
(**a**) Impact of different λ values on AUC results; (**b**) Impact of different λ values on precision results.

In both the LGC and LGC* methods, there is a key adjustable parameter λ. This is also referred to as the weight factor. In most similar research, the weight factor is commonly defined in the interval [0, 1]. This definition allows it to balance two or more terms without altering other factors, often yielding favorable results. We can observe from Figure 3 that, in most of the networks, as the parameter λ increases from 0.5 to 0.8, the values of AUC and precision slowly increased. When the parameter λ exceeded 0.8, these 10 networks started to either remain constant or exhibit a slight decreasing trend. Based on this observation, it is believed that the optimal performance of the LGC and LGC* algorithms is achieved when the adjustable parameter λ is set at 0.8. It can be thus inferred that assigning higher weights (λ = 0.8) to local features in these algorithms can more effectively predict links between networks. Moreover, through experimentation, we found that by squaring the values in the local proximity algorithm for nodes, there was a further enhancement in link prediction metrics. This is because when calculating proximity and clustering coefficients, the squaring operation amplifies those values that are already high and diminishes those that are low. This results in nodes with higher proximity and clustering coefficients having similarity scores that are more distinctively differentiated from other nodes. We refer to this optimized LGC algorithm as LGC*.

Table 4 displays a comparison of the AUC values between LGC, LGC*, and other link prediction algorithms. An analysis of the LGC algorithm shows that it achieved the highest AUC values in the LESM and Karate networks. Notably, the PA algorithm performed significantly worse in the Football network, suggesting that the PA algorithm might not be suitable for link prediction in smaller networks. In contrast to the PA algorithm, LGC exhibited superiority in smaller networks. In the Jazz network, the AUC value of the LGC algorithm reached 0.9627, while the improved LGC* raised the AUC to 0.9684. LGC, when compared to the LP algorithm in the USAir network, showed an improvement of 5 percentage points. On further examination of the optimized LGC*, it was observed that, compared to the original LGC, LGC* further enhanced performance by an average increase of 0.5 percentage points in AUC values. In summary, LGC achieves superior AUC values in many networks, while LGC* further amplifies the strengths of LGC.

In comparison to the precision values of other link prediction algorithms, as shown in Table 5, our proposed LGC algorithm achieved the best precision values in the USAir, POL, and Karate networks. In other networks, the precision values of the LGC algorithm are commendable, just slightly below the highest values. In the USAir network, the precision of the LGC algorithm was 3.3 percentage points higher than the best values of other algorithms. CCLP algorithm is similar to LGC in considering the factor of clustering coefficient, but LGC predicts more effectively according to our experiments. Lastly, looking at the optimized LGC*, it is evident from Table 5 that (1) apart from the Jazz, LESM, Route, and STMA networks, LGC* achieved the best precision values compared to the other six networks and (2) consistent with the AUC analysis, LGC* achieved higher precision value than LGC. In a nutshell, compared to traditional local similarity indicators such as CN and AA, and global similarity indicators like LP, Katz, both LGC and LGC* algorithms demonstrated superior predictive accuracy. 

### 3.4. Complexity Analysis

To test algorithm efficiency, let the number of nodes in the network be n, and the average node degree be d. The CN algorithm first needs to search for each pair of nodes to be predicted in the network, and then find the common neighbors between these two nodes. Therefore, the time complexity of the CN algorithm is $O\left(n^{2}\right)$. The AA and RA algorithms, based on the common neighbors, perform some calculations according to the degrees of the nodes. Thus, their time complexity is the same as that of the CN algorithm.

For neighbor-based methods (such as AA and RA), this type of algorithm needs to consider the potential common neighbors for each pair of nodes, which adds complexity related to the average node degree d. However, the PA algorithm is based only on the degree of nodes and does not need to consider the relationships between neighbors, so its complexity is lower.

The overall complexity of LGC and LGC* algorithms is determined by the maximum complexity among these functions. The most time-consuming operations in these algorithms are calculating the average shortest path in the network and calculating similarity. To calculate the shortest paths between all pairs of nodes, the worst-case time complexity is $O\left(n^{3}\right)$ (for example, when using the Floyd–Warshall algorithm). 

### 3.5. Robustness Analysis

In complex networks, robustness is crucial for the successful application of link prediction algorithms. To comprehensively assess the performance stability of the LGC algorithm under different network structures and data distributions, this study delved deeply into its robustness.

**Figure 4:**
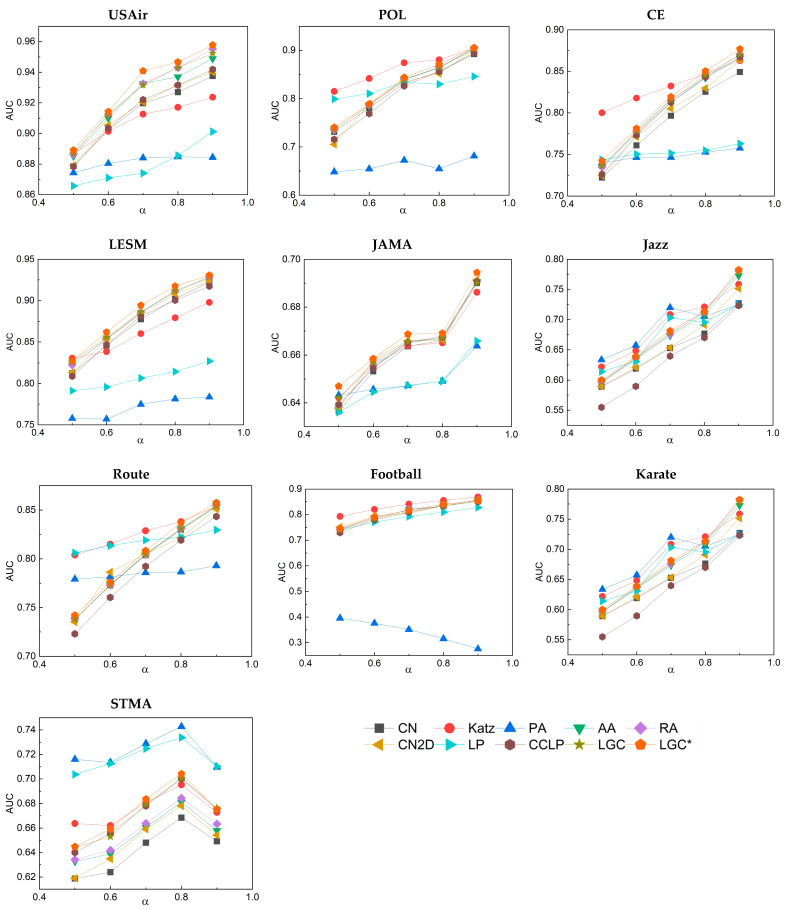
AUC results correspond to different proportions of the training set.

**Figure 5:**
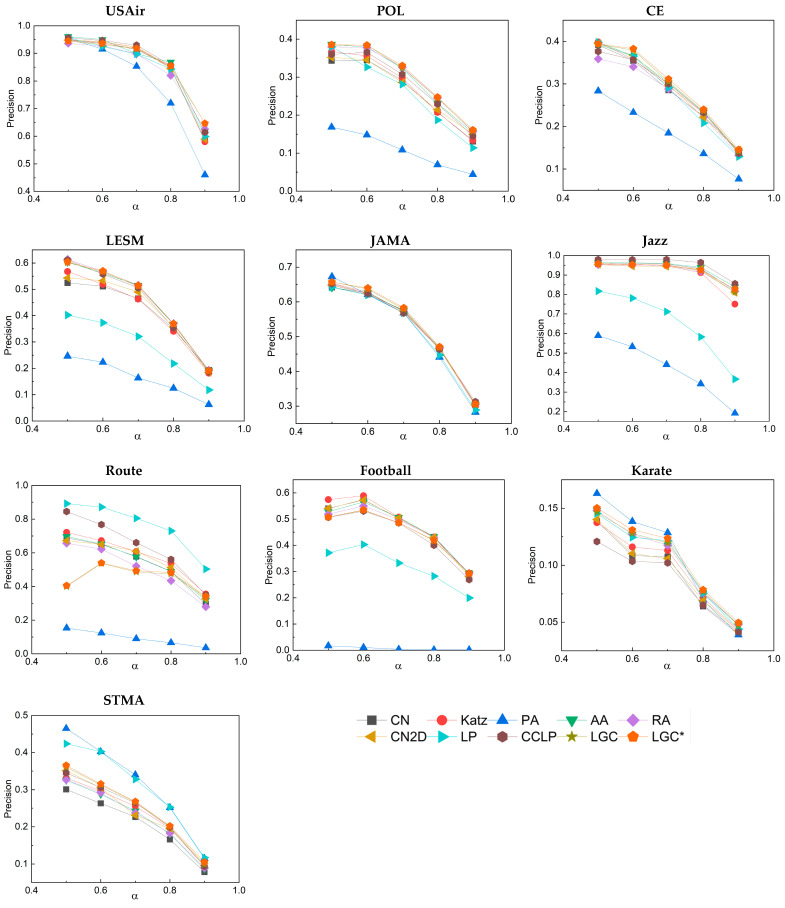
Precision results corresponding to different proportions of the training set.

First, we partitioned the training data differently and examined the performance when the training set was divided into ratios of 50%, 60%, 70%, 80%, and 90%. The corresponding results are displayed in Figure 4 and Figure 5. Notably, even with a reduced volume of training data, the LGC algorithm still maintained a relatively high AUC value, demonstrating its high robustness. Concurrently, through the precision variation curve in Figure 5, we further confirmed that the LGC algorithm maintained robust predictive accuracy under different training set ratios.

Unlike the AUC metric, most evaluation metrics exhibited better precision results under a higher proportion of test set $E_{P}$. This is primarily attributed to the increase in the number of connected edges that can be correctly detected as the size of the test set expands, thereby making it easier to discover missing edges. A further analysis revealed that both LGC and LGC* generally outperformed other classical link prediction algorithms in terms of AUC and precision and maintained stability under various training set divisions. This stability is the result of the LGC algorithm combining the local and global features of the network and making full use of the topological information.

In summary, the LGC algorithm not only surpasses most traditional methods in terms of predictive accuracy but also demonstrates evident advantages in terms of robustness. These results provide strong support for the practical application of the LGC algorithm and prove its potential as an effective link prediction tool.

## 4. Discussion and Conclusions

This study proposed a novel link prediction algorithm, named the Link Prediction Algorithm Based on Local and Global Proximity Weighting (LGC), along with its further optimized version, LGC*. Distinct from the current mainstream link prediction algorithms, LGC and LGC* comprehensively consider the proximity of nodes and the clustering coefficient of their common neighbors. This approach thoroughly exploits both local and global features of the network, thereby more accurately measuring the similarity between nodes. To validate its effectiveness, experiments were conducted on 10 real-world datasets, and the results indicate that, compared with the highest AUC and precision values of other methods, the LGC* algorithm shows an average improvement of one percentage point. The experiments have confirmed the superior performance of both the LGC and LGC* algorithms.

The LGC algorithm not only provides researchers with a new method to predict potential links in networks, serving as a powerful tool for various practical applications such as social network friend recommendations and functional gene predictions in biological networks, but also introduces a fresh research perspective to the entire field of link prediction.

Despite the superior performance of LGC and LGC* in multiple experiments, they still face several challenges. The most apparent issue is their high time complexity, which may limit their application in large-scale networks. To further improve the efficiency and scalability of the algorithms, future work will focus on their refinement and optimization, aiming to reduce the time and space overhead while maintaining high predictive accuracy. Additionally, plans are in place to conduct more extensive experiments on additional real-world datasets, in order to further validate and refine the algorithms’ practical applications.

## Figures and Tables

**Table 1:** Symbol definitions.

Symbols	Definitions
$S_{xy}$	Similarity Score of Node Sum
β	Weight Factor
$k_{x}$	Degree of a Node x
$k_{z}$	Degree of a Node z
$\Gamma \left(x\right)$	Neighbor Set of a Node x
$C_{Z}$	Clustering Coefficient of a Node z
$\Gamma \left(x\right)\cap \Gamma \left(y\right)$	Common Neighbors of x and y

**Table 2:** Similarity definition of classical algorithms.

Algorithm Acronym	Definitions
CN	$\begin{array}{c} S_{xy}=\left|\Gamma \left(x\right)\cap \Gamma \left(y\right)\right| \end{array}$
Katz	$S_{xy}={\left(I-\beta A\right)}^{-1}-I$
PA	$S_{xy}=k_{x}*k_{y}$
AA	$S_{xy}\begin{array}{c} =\underset{z\in \Gamma \left(x\right)\cap \Gamma \left(y\right)}{\sum }\frac{1}{\log k_{z}} \end{array}$
RA	$S_{xy}\begin{array}{c} =\underset{z\in \Gamma \left(x\right)\cap \Gamma \left(y\right)}{\sum }\frac{1}{k_{z}} \end{array}$
CN2D	$\begin{array}{c} S_{xy}=\\ \left|\Gamma \left(x\right)\cap \Gamma \left(y\right)\right|+\beta \left(\frac{1}{\max\left(k_{x},k_{y}\right)}\underset{z\in \Gamma \left(x\right)\cap \Gamma \left(y\right)}{\sum }\left|\Gamma \left(z\right)\right|\right) \end{array}$
LP	$S_{xy}\begin{array}{c} =\left|\frac{\Gamma \left(x\right)\cap \Gamma \left(y\right)}{k\left(x\right)*k\left(y\right)}\right| \end{array}$
CCLP	$\begin{array}{c} S_{xy}=\underset{z\in \Gamma \left(x\right)\cap \Gamma \left(y\right)}{\sum }C_{Z} \end{array}$

**Table 3:** Statistical information of 10 real-world networks.

Networks	*N*	*M*	$\langle c\rangle$	$\langle k\rangle$	*D*
USAir	332	2162	0.749	12.807	0.039
POL	105	441	0.487	8.400	0.081
CE	297	2148	0.308	14.465	0.053
LESM	77	254	0.735	6.597	0.087
JAMA	62	1187	0.667	37.645	0.617
Jazz	198	2742	0.618	27.697	0.141
Route	2113	6632	0.123	3.139	0.003
Football	115	613	0.403	10.66	0.309
Karate	34	78	0.571	4.588	0.256
STMA	54	350	0.413	12.963	0.245

**Table 4:** Comparison of AUC results of LGC algorithm and benchmarks.

Network	CN	Katz	PA	AA	RA	CN2D	LP	CCLP	LGC	LGC*
USAir	0.9375	0.9237	0.8844	0.9488	0.9552	0.9401	0.9012	0.9418	0.9523	0.9577
POL	0.8923	0.9044	0.6811	0.9025	0.9047	0.9005	0.8460	0.8944	0.9031	0.9082
CE	0.8491	0.8628	0.7576	0.8658	0.8704	0.8631	0.7631	0.8670	0.8701	0.8766
LESM	0.9225	0.8978	0.7837	0.9275	0.9276	0.9231	0.8271	0.9173	0.9284	0.9305
JAMA	0.6900	0.6862	0.6637	0.6907	0.6910	0.6914	0.6658	0.6906	0.6901	0.6954
Jazz	0.9521	0.9383	0.7655	0.9593	0.9656	0.9575	0.8370	0.9561	0.9627	0.9684
Route	0.8532	0.8539	0.7929	0.8545	0.8543	0.8511	0.8296	0.8434	0.8543	0.8674
Football	0.8534	0.8693	0.2758	0.8543	0.8545	0.8569	0.8272	0.8499	0.8501	0.8543
Karate	0.7271	0.7584	0.7250	0.7725	0.7820	0.7514	0.7248	0.7231	0.7776	0.7823
STMA	0.6493	0.6728	0.7097	0.6579	0.6633	0.6542	0.7103	0.6750	0.6765	0.6851

**Table 5:** Comparison of precision results of LGC algorithm and benchmarks.

Network	CN	Katz	PA	AA	RA	CN2D	LP	CCLP	LGC	LGC*
USAir	0.5850	0.5800	0.4605	0.6070	0.6255	0.5891	0.5989	0.6145	0.6465	0.6475
POL	0.1310	0.1340	0.0440	0.1535	0.1565	0.1496	0.1140	0.1455	0.1575	0.1610
CE	0.1405	0.1415	0.0765	0.1400	0.1320	0.1450	0.1290	0.1365	0.1410	0.1457
LESM	0.1935	0.1820	0.0625	0.1930	0.1930	0.1847	0.1180	0.1875	0.1895	0.1913
JAMA	0.3050	0.3000	0.2825	0.3050	0.3055	0.3041	0.2890	0.3125	0.3030	0.3161
Jazz	0.8210	0.7500	0.1920	0.8390	0.8155	0.8089	0.3665	0.8560	0.8105	0.8275
Route	0.2960	0.3550	0.0360	0.3185	0.2795	0.3265	0.5035	0.3500	0.3335	0.3412
Football	0.2935	0.2895	0.0015	0.2935	0.2935	0.2815	0.1995	0.2695	0.2881	0.2948
Karate	0.0395	0.0430	0.0390	0.0475	0.0480	0.0425	0.0440	0.0415	0.0482	0.0496
STMA	0.0780	0.0940	0.1150	0.0860	0.0890	0.0961	0.1170	0.0935	0.0990	0.1053

## Data Availability

Not applicable.
